# Neurochemical Changes in the Mouse Hippocampus Underlying the Antidepressant Effect of Genetic Deletion of P2X7 Receptors

**DOI:** 10.1371/journal.pone.0066547

**Published:** 2013-06-21

**Authors:** Cecilia Csölle, Mária Baranyi, Gabriella Zsilla, Ágnes Kittel, Flóra Gölöncsér, Peter Illes, Edit Papp, E. Sylvester Vizi, Beáta Sperlágh

**Affiliations:** 1 Department of Pharmacology, Institute of Experimental Medicine, Hungarian Academy of Sciences, Budapest, Hungary; 2 Department of Cellular and Network Neurobiology, Institute of Experimental Medicine, Hungarian Academy of Sciences, Budapest, Hungary; 3 Department of Pharmacology and Pharmacotherapy, Semmelweis University, Budapest, Hungary; 4 Rudolf-Boehm-Institute of Pharmacology and Toxicology, University of Leipzig, Leipzig, Germany; Centre national de la recherche scientifique, University of Bordeaux, France

## Abstract

Recent investigations have revealed that the genetic deletion of P2X7 receptors (P2rx7) results in an antidepressant phenotype in mice. However, the link between the deficiency of P2rx7 and changes in behavior has not yet been explored. In the present study, we studied the effect of genetic deletion of P2rx7 on neurochemical changes in the hippocampus that might underlie the antidepressant phenotype. P2X7 receptor deficient mice (P2rx7−/−) displayed decreased immobility in the tail suspension test (TST) and an attenuated anhedonia response in the sucrose preference test (SPT) following bacterial endotoxin (LPS) challenge. The attenuated anhedonia was reproduced through systemic treatments with P2rx7 antagonists. The activation of P2rx7 resulted in the concentration-dependent release of [^3^H]glutamate in P2rx7+/+ but not P2rx7−/− mice, and the NR2B subunit mRNA and protein was upregulated in the hippocampus of P2rx7−/− mice. The brain-derived neurotrophic factor (BDNF) expression was higher in saline but not LPS-treated P2rx7−/− mice; the P2rx7 antagonist Brilliant blue G elevated and the P2rx7 agonist benzoylbenzoyl ATP (BzATP) reduced BDNF level. This effect was dependent on the activation of NMDA and non-NMDA receptors but not on Group I metabotropic glutamate receptors (mGluR_1,5_). An increased 5-bromo-2-deoxyuridine (BrdU) incorporation was also observed in the dentate gyrus derived from P2rx7−/− mice. Basal level of 5-HT was increased, whereas the 5HIAA/5-HT ratio was lower in the hippocampus of P2rx7−/− mice, which accompanied the increased uptake of [^3^H]5-HT and an elevated number of [^3^H]citalopram binding sites. The LPS-induced elevation of 5-HT level was absent in P2rx7−/− mice. In conclusion there are several potential mechanisms for the antidepressant phenotype of P2rx7−/− mice, such as the absence of P2rx7-mediated glutamate release, elevated basal BDNF production, enhanced neurogenesis and increased 5-HT bioavailability in the hippocampus.

## Introduction

Mood disorders, including major depressive disorder (MDD) and bipolar disorder (BPD), are the most common psychiatric illnesses [1], [2], which are caused by complex interactions between genes, developmental and environmental factors. Genetic research has identified several chromosomal regions and genes involved in the susceptibility to mood disorders, but no clear etiological mechanism has been determined. Previous studies revealed mutations in the gene encoding the P2X7 purinergic receptor (P2rx7), which are associated with the development of MDD and BPD [3], [4], [5], [6], [7], [8]. Although subsequent studies have failed to confirm this association [9], [10], [11], more recent data have again reaffirmed the potential of these polymorphisms to affect the pathology of mood disorders [12], [13]. In addition, a clinical study showed the reduced mRNA expression of the P2X7 receptor in a set of patients suffering from depression and posttraumatic stress disorder characterized by the increased risk of suicide [14]. P2rx7 belongs to the family of ionotropic P2X receptors that are sensitive to ATP and other related nucleotides. These receptors are distributed in hematopoietic cells, epithelial cells, neurons, astrocytes, oligodendrocytes and microglia. P2rx7 plays an important role in the processing and secretion of mature pro-inflammatory cytokines, such as interleukin (IL)-1β, IL-18, and tumor necrosis factor (TNF)-α, and in ATP-mediated apoptosis. The primary role of P2rx7s in the brain is regulation of neurotransmitter release [15], [16], [17]. The activation of P2rx7 results in Ca^2+^ influx [18], increased glutamate and GABA release from brain slices [19], [20] and nerve terminals [21], [22].

Previous studies have demonstrated that the genetic deletion and pharmacological antagonism of P2rx7 leads to an antidepressant phenotype in several behavioral models [23], [24], [25]. We found that P2rx7−/− mice displayed a decreased immobility response in the forced swim (FST) and tail suspension (TST) tests, which can be reproduced by subacute administration of the selective P2rx7 antagonist Brilliant Blue G (BBG). We also presented evidence that the deletion of P2rx7 in non-hematopoietic cells leads to the observed antidepressant phenotype. When bone marrow chimeras were generated that lacked the P2rx7 only in their hematopoietic compartment, no difference was found in behavioral tests, indicating that the antidepressant phenotype found in P2rx7−/− mice was not transferred to wild-type recipients with the engraftment of the P2rx7−/− bone marrow cells [25]. Consequently, the expression of P2rx7 in other cell types, particularly neurons or astrocytes, might be responsible for the associated changes in mood. In addition, we found that the deficiency of P2X7 receptors leads to a widespread alteration of the gene expression in the limbic system, including the up and downregulation of genes crucial for synaptic transmission and plasticity, such as glutamatergic and GABAergic receptor subunits [25]. Consistent with these results, other studies have shown enhanced c-Fos expression in the hippocampus of P2rx7−/− mice after repeated forced swim tests, which indicates that the hippocampus is an important target area that mediates the effect of P2rx7 activation on emotional behavior [24].

Despite these data, the key question how the activity of P2X7 receptor leads to alterations in animal behavior remains unknown. It has been repeatedly shown that major depression is characterized by a reduction of neuronal plasticity and a 2-week administration of antidepressant treatment is sufficient to normalize this deficit and generate a neuroadaptive mechanism that might enhance neuronal plasticity [26], [27]. Neurotrophins, such as brain-derived neurotrophic factor (BDNF), promote neuronal survival and enhance synaptic plasticity [28]. BDNF is synthesized as a 30–35-kDa-precursor protein, which is proteolytically cleaved to produce a mature and functional enzyme that plays a crucial role in the development, differentiation and survival of neuronal populations within the central and peripheral nervous system. Several studies have demonstrated a strong association between the expression of BDNF and the onset of depression. Reduced BDNF mRNA and protein expression have been detected in the hippocampus of postmortem brains from suicide victims [29] and serum BDNF levels are reduced in depressed patients [30]. Consistent with these findings, it has been shown that the infusion of BDNF into the brain results in an antidepressant-like phenotype [31]. However, the lack of BDNF is not sufficient to produce a depressive phenotype, although it is required for the behavioral response to antidepressants [32].

As a consequence of an alteration in the level of neurotrophic factors, or independently from it, an aberrant regulation of adult neurogenesis in the hippocampus has also been implicated in the pathophysiology of MDD. Human in vivo imaging studies have demonstrated a reduction in the volume of the hippocampus in depressed patients [33], and this effect can be ameliorated through antidepressant treatment [34]. Post-mortem studies also verified a decrease in the size and/or number of neuronal cell bodies in this brain area [35]. Studies in rodents have consistently shown that stress is one of the most powerful stimuli that leads to a decrease in adult neurogenesis in the hippocampus [36]. Moreover, chronic, but not acute antidepressant treatment normalizes neurogenesis in the adult hippocampus, and the inhibition of neurogenesis prevents the behavioral response to antidepressants, providing a direct link between neurogenesis and the antidepressant response [37].

Nevertheless, other mechanisms, such as the alteration in extracellular levels of monoamines in the brain might also be responsible for the characteristic action of antidepressant drugs observed in rodent models of depression [38].

In the present study, to gain insight into the cellular basis of differential behavioral responses, we used a P2rx7−/− mouse model to demonstrate that (1) the genetic deletion of P2rx7 induced neurochemical changes characteristic of antidepressant treatment, such as the dysregulation of monoaminergic transmission and elevated BDNF levels in the hippocampus, and (2) the lack of P2rx7-mediated glutamate release and inhibition of BDNF production, resulting in an enhanced hippocampal neurogenesis is a potential mechanism for the antidepressant phenotype.

## Materials and Methods

### Animals

This study was performed in strict accordance with the recommendations in the Guide for the Care and Use of Laboratory Animals of the National Institutes of Health and the local Animal Care Committee of the IEM HAS approved all experimental procedures (Permission No: 22.1/3671/003/2008). This study used 2- to 3-months old (approx. 30 g) male wild type (P2rx7+/+), and P2rx7 knockout (P2rx7−/−) mouse littermates, which were housed under a 12 h on/12 h off light cycle with 60±10% humidity in a temperature-controlled room (23±2°C). Food and water were supplied ad libitum. Homozygous P2rx7−/− mice were bred to C57BL/6 mice. Christopher Gabel (Pfizer Inc., Groton, CT, USA) kindly supplied the original breeding pairs of P2rx7−/− mice (C57BL/6J based). An overall six backcrosses on C57BL/6 were performed for the P2rx7 knockout mouse colony used in our experiments. Offspring of this mouse line were cross-bred with P2rx7+/+ mice and the resulting heterozygotes were used as breeding stock for the F1 generation offspring employed in the behavior studies. The animals contained the DNA construct (P2X7-F1 (5′-CGGCGTGCGTTTTGACATCCT-3′) and P2X7-R2 (5′-AGGGCCCTGCGGTTCTC-3′)), which have been previously shown to generate the genetic deletion of P2rx7 [39]. The animals were genotyped using PCR analysis as described earlier [39].

### Behavior Experiments

#### Automated Tail Suspension Test (TST)

All experiments and treatments were performed during the light phase (7.00 AM–7.00 PM). The TST was conducted using an automated tail suspension device (BIO-TST2, Bioseb, France) in accordance with a previously described method [40]. The device consisted of a single tail suspension module with three chambers, connected to a personal computer that recorded the activity of the animals during the test sessions. Three mice were individually suspended by the tail onto the hooks of the device using adhesive tape (distance from tip of tail was 1–2 cm). The measurements were automatically recorded within 5–10 seconds after placing the last animal into the chamber, and each measurement lasted 6 min. During the test, the animals showed several escaping behaviors with temporary periods of immobility. The threshold level was set at 6. To examine endotoxin-induced depressive behavior, the animals were subjected to bacterial lipopolysaccharide injection (LPS, serotype 055:B5, Sigma, St. Louis, MA, USA, 1 mg/kg i.p.) at 24 h before a single 6-min test period. Brilliant blue G (BBG, 50 mg/kg i.p.) or an equal volume of saline was injected at 30 min before the test period. The time of immobility was expressed in sec. The P2rx7+/+ and P2rx7−/− mice were subjected to alternation testing. In accordance with the observations of Mayorga and Lucki (2001), some of the animals (0–16%, depending on the experiment) displayed tail-climbing behavior. The data of these animals were excluded from the calculations during the post hoc analysis.

#### Sucrose Preference Test (SPT)

The experiments were performed using the two-bottle sucrose preference paradigm [41]. The decreased preference for sucrose vs. water in this test has been proposed to reflect impaired sensitivity towards reward and to model anhedonia [42]. Mice were offered a choice of 2% sucrose solution or water during 12-hour nocturnal periods (starting at 19∶00). The bottles were weighed before and after each test session to monitor sucrose and water consumption. The relative position of the sucrose and water bottles were swapped every night to avoid place preferences. The baseline sucrose intake was measured under a habituation period of 3–4 days before administering the treatments, and the mean volume ingested over the last 3 training sessions was used as a baseline value. The sucrose consumption was measured for 4 consecutive nights after LPS (0.2 mg/kg i.p.) or saline administration and expressed as g/100 g body weight or as a percentage of the baseline (%). The LPS dosage was chosen based on preliminary experiments, which showed that a 0.2 mg/kg dose elicits a relatively selective anhedonia response.

### [^3^H]Glutamate ([^3^H]Glu) Release Experiments

The [^3^H]Glu release experiments were conducted using the method described in our previous papers [20]. Briefly, the mice were anaesthetized under light CO_2_ inhalation, and subsequently decapitated. The hippocampus was dissected from each mouse in ice-cold Krebs solution saturated with 95% O_2_ and 5% CO_2_, sectioned (400-µm-thick slices) using a McIlwain tissue chopper and incubated in 1 ml of modified Krebs solution (113 mM NaCl, 4.7 mM KCl, 2.5 mM CaCl_2_, 1.2 mM KH_2_PO_4_, 1.2 mM MgSO_4_, 25.0 mM NaHCO_3_, and 11.5 mM glucose), pH 7.4, in the presence of 5 µCi/ml [^3^H]glutamic acid ([^3^H]Glu, 9.8×10^−8^ M, specific activity 51 Ci/mmol; Perkin Elmer) for 45 min. The medium was bubbled with 95% O_2_ and 5% CO_2_ and maintained at 32°C. After loading, the slices were continuously superfused with 95% O_2_ and 5% CO_2_-saturated modified Krebs solution (flow rate: 0.7 ml/min).

Subsequently, the perfused samples were collected over a 3-min period and measured for tritium content. At 6 min after the start of the collection, the slices were subjected to a 6-min perfusion of the agonist (ATP) at various concentrations (1, 3, 6 and 10 mM) and then changed to normal Krebs solution until the end of the collection period. In some experiments, the P2X1 receptor antagonist, NF449 (100 nM), or the selective P2rx7 antagonist Brilliant blue G (100 nM) was applied to the perfusion solution at 15 min before the beginning of the ATP perfusion.

The radioactivity released from the preparations was measured using a Packard 1900 Tricarb liquid scintillation spectrometer, using Ultima Gold Scintillation cocktail. The release of tritium was expressed as a percentage of the amount of radioactivity in the tissue at the sample collection time (fractional release). The tritium uptake in the tissue was determined as the sum of release+the tissue content after the experiment and expressed in Bq/g. For the evaluation of the basal tritium outflow the fractional release measured in four consecutive 3 min samples under drug free conditions were taken into account. The ATP-induced [^3^H]Glu efflux calculated as the net release in response to the respective stimulus by subtracting the release before the stimulation from the values measured after stimulation. Selected samples collected under resting conditions and during the peak of the ATP-evoked response were subjected to further HPLC analyses.

### Analysis of NR2B mRNA Expression Using Quantitative Real-time PCR

P2rx7 wild type and knockout mice were anesthetized under light CO_2_ inhalation, and subsequently decapitated. The hippocampus from each mouse was dissected in ice-cold Krebs solution saturated with 95% O_2_ and 5% CO_2_, sectioned (400 µm thick slices) using a McIlwain tissue chopper and incubated in 2 ml of modified Krebs solution in the presence of 1 ml Hibernate medium (Invitrogen Life Technology, Grand Island, NY, USA), ascorbic acid (300 µM) and Na_2_EDTA (30 µM) for 60 min. The medium was bubbled continuously with 95% O_2_ and 5% CO_2_ and maintained at 37°C. The hippocampal slices were collected, frozen on dry ice and stored at −70°C until further investigation. Each experimental group contained 3–4 mice. Total RNA samples were isolated and purified from the cell lysates using the RNeasy Lipid Tissue Mini Kit (Qiagen) according to the manufacturer’s instructions. The RNA (2 µl) was reverse transcribed using the RevertAid First Strand cDNA Synthesis Kit (Fermentas, Vilnius, Lithuania) as described in our previous study [20], [43]. Briefly, 1 µg of total RNA reverse transcribed using 1 µl of RevertAid H Minus M-MuLV reverse transcriptase in a mixture containing 5 µl of 5X reaction buffer, 1 µl of random hexamer primer (10 pmol/µl), 1 µl of RiboLock™ RNase Inhibitor (20 u/µl), and 2 µl of 10 mM dNTP mix in a final volume of 20 µl with 0.1% diethylpyrocarbonate-treated distilled water. The reverse transcription reaction was performed at 70°C for 5 min, followed by incubation at 25°C for 5 min, synthesis at 25°C for 10 min, and a final incubation at 42°C for 60 min. The resulting cDNA samples were stored at −20°C. The expression level of the target gene was determined using the cDNA samples with quantitative real-time PCR (Rotor-Gene 3000; Corbett Research, Sydney, Australia). The Real-time PCR analysis was performed according to standard protocols using a LightCycler DNA Master SYBR Green I Kit (Roche, Indianapolis, IN, USA). The PCR conditions were optimized for primers, templates and MgCl_2_. The PCR cycling protocols was set to the following conditions: initial denaturation at 95°C for 10 min followed by 40 cycles at 94°C for 15 sec, 64°C for 30 sec, and 72°C for 10 sec. The PCR primers were based on previous study of Xiaoping Du et al. [44]. The following primers were used for mRNA detection: *NR2B* forward primer, 5′ GTG AGA GCT CCT TTG CCA AC; *NR2B* reverse primer, 5′ GTC AGG GTA GAG CGA CTT GC; *18S* forward primer, 5′-GTAACCCGTTGAACCCCATT, and *18S* reverse primer, 3′-CCATCCAATCGGTAGTAGCG.

#### Analysis of real-time PCR measurements

To ensure reaction specificity and accurate quantification, a melting curve analysis was performed after each reaction, which confirmed the lack of primer–dimer artifacts or contamination in all cases. All ΔCt values were calculated using Rotor Gene 5 software (Corbett Research, Sydney, Australia). The expression level of the target genes was normalized to the expression level of 18S rRNA as a reference or housekeeping gene. The target gene and the reference gene were measured together within the same experiment. The efficiency calibrated model of Pfaffl was applied to compare the expression level of target genes between the different experimental groups [45]. Differences in the gene expression levels between the experimental groups were considered significant when the *P* level was <0.05. Data are presented as the mean normalized expression ratio ± SEM.

### NR2B Immunostaining

Male P2rx7+/+ or P2rx7−/− mice (62–65 days old, appr. 30 g) from our in-house colony were used. Animals were deeply anesthetized and perfused transcardially with 4% paraformaldehyde in 0.1 M phosphate buffer (PB, pH 7.4). Brains were removed and post-fixed overnight at 4°C. The block containing the whole hippocampus was dissected out and 100 µm coronal sections were serially sectioned with a Leica vibratome and immersed free-floating in 0.1 M PB. After extensive washing, sections were treated with 0.2M HCl containing 0.2 mg/ml pepsin at 37°C for 15 min, then rinsed in PB three times and washed for 3X10 min in 0.1M Tris buffer (TBS). Nonspecific binding sites were blocked by 10% normal horse serum (Vector Laboratories, Burlingame, CA) for 2 hours and incubated with a mouse anti-NR2B (GluN2B/NR2B antibody (UC Davis/NIH NeuroMab Facility) diluted 1∶1000 in TBS containing 2% normal horse serum for 24 h at 4°C. After washing in TBS three times (10 min each), anti-mouse Cy3 (Jackson ImmunoResearch Europe Ltd. Suffolk CB8 1JX, UK) secondary antibody was applied in 1∶1000 for 2 hours at room temperature. Sections were washed in TB, mounted on polylysine-coated slides and cover slipped with Vectashield (Vector Laboratories). Specificity of NeuroMab GluN2B/NR2B antibody was validated on KO tissues and it did not cross react with glutamate receptors NR2A, NR2C or NR2D (see information http://catalog.antibodiesinc.com/item/neuromabs/receptors/75-097?&plpver=10&origin=advsrch&by=prod&filter=0&categid=1025&prodid=1028).

Likely due to the HCl-pepsin treatment, careful blocking with 10% NHS and 2% NHS content in the first and second antibody solutions could not prevent some background staining in blood vessels. However, the similar intensity of this staining in P2rx7+/+ and P2rx7−/− hippocampal sections can illustrate the same image acquisition and editing parameters.

Confocal images were acquired at the same depth of the sections at same acquisition parameters with a Nikon A1R confocal system on an inverted Nikon Ti-E microscope (objective 20X DIC N1, numerical aperture 0.45 for large images and Plan Apo VC 60× Oil DIC N260X, numerical aperture 1.4 for details) equipped with NIS-Elements C software. Images were edited, brightness as well as contrast were adjusted using Adobe Photoshop CS3 (San Jose, CA, USA). The average intensity of NR2B immunostaining was quantified with NIH ImageJ program (U.S. National Institutes of Health, Bethesda, MD).

### BDNF Protein Assay

#### Ex vivo study

The P2rx7 wild type and knockout mice received an intraperitoneal injection of sterile saline (0.9% NaCl) or LPS from *E. Coli* (Sigma, 055:B5; 1 mg/kg; 0.1 ml/mouse) and decapitated at 24 h later. The hippocampus was collected, frozen on dry ice and stored at −70°C until further investigation. Each experimental group contained 4–6 mice.

#### In vitro study

The P2rx7 wild type and knockout mice were anesthetized under light CO_2_ inhalation and decapitated. The hippocampus was dissected in ice-cold Krebs solution saturated with 95% O_2_ and 5% CO_2_, sectioned (400 µm thick slices) using a McIlwain tissue chopper and incubated in 2 ml of modified Krebs solution in the presence of 1 ml Hibernate medium [28], ascorbic acid (300 µM) and Na_2_EDTA (30 µM) for 60 min. The medium was bubbled continuously with 95% O_2_ and 5% CO_2_ and maintained at 37°C. The P2rx7 agonist, BzATP, was added to the incubation solution for 30 min, whereas BBG was applied 15 min before BzATP application. RO-256981, 6-cyano-7-nitroquinoxaline-2,3-dione (CNQX) and 3-Chloro-4-fluoro-N-[4[[2(phenylcarbonyl)hydrazino]carbonyl]benzyl] benzenesulfonamide (TCN-201) were applied for 15 min, while MCPG was administered for 30 min. The group I metabotropic glutamate receptor agonist, dihydroxyphenylglycine (DHPG), was administered for 10 min. The DHPG, MCPG, and RO-256981 dosages were chosen based on previous studies [46], [47], [48]. In the experiments with BzATP, Mg^2+^ was omitted from the incubation solution. The hippocampal slices were collected, frozen on dry ice and stored at −70°C until further investigation. Each experimental group contained 3–4 mice.

#### BDNF protein measurement

At the time of analysis, the samples were removed from the freezer and weighed. The BDNF extraction procedure was performed in accordance with the methods of a previous study [49]. For the BDNF assays, 2 ml lysis buffer (100 mM PIPES, pH 7, 500 mM NaCl, 0.2% Triton X-100, 0.1% NaN_3_, 2% BSA, 2 mM EDTA, 200 µM PMSF, 10 µg/mL aprotinin, 1 µg/mL leupeptin, and 0.5 mM sodium vanadate) was added to each sample, and the hippocampus was sonicated at power level 2 using pulses at 1 sec intervals for 10–15 sec. Subsequently, the samples were centrifuged at 16,000 × *g* for 30 min at 4°C. 100 µl aliquots of the resulting supernatants were removed and diluted with 400 µl of DPBS buffer (137 mM NaCl, 2.68 mM KCl, 1.47 mM KH_2_PO_4_, 8.1 mM Na_2_HPO_4_ (pH 7.35), 0.9 mM CaCl_2_·H_2_O, and 0.5 mM MgCl_2_·H_2_O). The supernatants were collected and stored at −70?C until further analysis. For the total free BDNF measurement, the samples were acid treated with 1 µl of 1 N HCl for each 50 µl of diluted samples to decrease the pH to 2.5, followed by incubation at room temperature for 15 min. The samples were neutralized with 1 µl of 1 N NaOH for each 50 µl of diluted samples. The levels of BDNF (both precursor and mature forms) expression were evaluated using an enzyme-linked immunosorbent assay (ELISA) kit and a BDNF Emax ImmunoAssay System (Promega Corporation, Madison, WI, USA), which is specific for mouse BDNF protein, according to the manufacturer’s instructions. The BDNF levels were calculated by plotting the optical density (OD) of each sample against the standard curve. A seven-point standard curve using two-fold serial dilutions in Reagent Diluent (according the manufacturer’s instructions) and a high standard of 500 pg/ml were used for the determination of BDNF levels. The assay detection limits were <15 pg/ml. Absorbance was measured at 450 nm using a Perkin-Elmer Victor 3V 1420 Multilabel Counter.

### 5-Bromo-2-deoxyuridine (BrdU) Incorporation Experiment

#### BrdU staining procedure

Male P2rx7+/+ and P2rx7−/− mice were used. The animals received intraperitoneal injections of 150 µl (3×50 mg/kg body weight) BrdU Labeling Reagent (Invitrogen) for 3–5 consecutive days. The animals were anesthetized and sacrificed on the day after the last injection of BrdU. The animals were perfused transcardially with 4% paraformaldehyde in 0.1 M phosphate buffer (PB, pH 7.4). The brains were removed and post-fixed overnight at 4°C. The entire hippocampus was dissected, and 40 µm coronal sections were serially sectioned using a Leica vibratome and immersed free-floating in 0.1 M PB. A one-in-six series of sections from every animal was used for the cell counts. The sections were washed (3×5 min) in 0.1 M PBS (pH 7.4). Peroxidase labeling was used to assess BrdU incorporation, therefore we applied 1% H_2_O_2_ for 30 min at RT to destroy endogenous peroxidase activity. After subsequent washing in PBS, the sections were transferred to 2 N HCl for 30 min at 37°C for DNA denaturation, rinsed in borate buffer (twice 10 min each), followed by a final rinse in PBS. The sections were incubated with a mouse anti-BrdU (Sigma, B8434) antibody diluted 1∶2000 in PBS containing 3% normal horse serum and 1% Triton X-100 for 24 h at 4°C. The ImmPRESS Universal Antibody Kit was used according to the manufacturer’s instructions (Vector Laboratories, Burlingame, CA) and diaminobenzidine with Ni intensification (DAB-Ni) was used as the chromogen. The sections were dried on glass slides, cleared with xylene and coverslipped with Depex (Sigma, Aldrich Co, St. Louis, MO, USA).

#### BrdU-positive cell counting

To compare the number of BrdU-positive cells in the rostral hippocampus of P2rx7+/+ and P2rx7−/− mice, a one-in-six series of coronal sections (200 µm apart) were drawn using a camera lucida with 20× objective. BrdU-positive (BrdU +) cells were counted in the granule cell layer, including the subgranular zone (defined as the 50 µm zone adjacent to the inner edge of the granule cell layer). The same areas and number of sections were investigated in each mouse, and we considered a cell as BrdU positive if the nucleus was completely filled with DAB-Ni endproduct. The result was expressed as the average number of BrdU-positive cells in a hippocampal section. Bright field microscopy was performed using a Zeiss microscope equipped with 20× objective lens for bright field imaging. The unpaired Student’s t-test with Welch’s correction was applied as a statistical test.

### HPLC Determination of Endogenous Noradrenaline (NA), 5-hydroxytryptamine (5-HT) and Glutamate (Glu)

The animals were sacrificed by decapitation, and the hippocampus was dissected and frozen in liquid nitrogen. The frozen tissue was weighed and homogenized in ice-cold 0.1 M perchloric acid containing 10 µM theophylline as internal standard and 0.5 mM sodium metabisulfite. The suspension was centrifuged at 300×*g* for 10 min at 4°C. The perchloric anion was precipitated using 1 M KOH and separated through centrifugation. The protein content of the pellet fraction was determined according to the method of Lowry [50]. The supernatant was stored at −20°C until further analysis. The biogenic amines were measured using two-dimensional reversed-phase and ion-pair reversed-phase chromatography as previously described [51] using a Gilson liquid chromatographic system (Gilson Medical Electronics Inc., Middletown, WI, USA) equipped with an Applied Biosystems 785/A UV and BAS CC-4 amperometric detector in a cascade line. The separations were performed on a 3 µm Discovery C18 HS (150×4.0 mm) analytical column, and the biogenic amines were measured at a 0.73-V potential electrochemical detection. The retention order of monoamines was NA 14.7 min, 3,4-dihydroxyphenylacetic acid (DOPAC) 16.8 min, 5-hydroxy indoleacetic acid (5-HIAA) 19.3 min, dopamine (DA) 25.5 min, homovanillic acid (HVA) 26.8 min, 3-methoxytyramine (3-MT) 31.2 min and 5-HT 39.3 min.

The separation of pre-column dansylated amino acids was performed using a gradient elution-working mode at ambient temperature. The mobile phase A consisted of 5/95 (v/v) 78/22 acetonitrile/methanol in 15 mM ammonium formate buffer, and the mobile phase B was composed of 90/10 (v/v 78/22) acetonitrile/methanol in ammonium formate buffer, pH 3.7. The mobile phase B increased linearly (at 0.11 min to 50% 17 min to 72% and 26 min to 100%, followed by a final run to 54 min), the flow rate was 0.7 ml/min. The analytical and the trap column were equilibrated for 10 min, and enrichment and clean-up procedures were performed. The dansylated derivatives were detected using an absorbance detector (Agilent 1100) at a 319 nm wavelength. The retention time of the dansylated Glu was 11.4 min. Pre-column derivatization was performed by mixing 50 µl of dansyl chloride prepared by dissolving 5 mg 5-(dimethylamino)naphthalene-1-sulfonyl chloride in acetonitrile daily and adding 50 µl of 2 M sodium carbonate, which contained norvaline (20 µM) as an internal standard in 25 µl of sample. After a 10-min reaction time at 60°C, the mixture was acidified using 25 µl of 6 M formic acid and injected onto the “trap-column”.

The concentrations of the separated compounds were calculated using a two-point calibration curve internal standard method: (Ai * f * B)/(C * Di * E) (Ai: Area of component; B: Sample volume; C: Injection volume; Di: Response factor of 1 pmol of standard; E: Protein content of sample; f: factor of Internal Standard (IS area in calibration/IS area in actual)). The data were expressed as pmol/mg protein (NA, 5-HT) or pmol per 3 min sample (Glu).

### [^3^H]NA and [^3^H]5-HT Release Experiments

[^3^H]NA and [^3^H]5-HT release was studied as described previously with small modifications [43], [52]. Briefly, mice were anaesthetized under light CO_2_ inhalation and decapitated. The hippocampi were dissected in ice-cold Krebs’ solution saturated with 95% O_2_ and 5% CO_2_, and 400 µm thick slices were prepared. Slices were incubated for 45 min in 1 ml of modified Krebs’ solution saturated at 95% O_2_ and 5% CO_2,_ and containing [^3^H]5-HT or [^3^H]NA (10 and 5 µCi/ml, specific activity 24 and 30 Ci/mmol, respectively, Amersham, Little Chalfont, UK), ascorbic acid (300 µM) and Na_2_EDTA (30 µM). Thereafter, slices were transferred to tissue chambers, and preperfused for 1 hour (flow rate: 0.65 ml/min) with modified Krebs’ solution. In [^3^H]5-HT release experiments, the medium also contained the serotonin reuptake inhibitor citalopram (300 nM). After washing, 3 min perfusate samples were collected and assayed for [^3^H]5-HT/[^3^H]NA. At 6 and 36 min, two identical periods of electrical field stimulations (S1, S2; 25 V, 1 msec, 2 Hz, 240 shocks) were delivered by a Grass S88 stimulator (Grass Instruments, Quincy, MA, USA). The temperature was maintained at 37°C throughout the experiment. The radioactivity released from the preparations was measured as described above ([^3^H]Glu release experiments) and tritium efflux was expressed in becquerel per gram (Bq/g). Electrical stimulation-induced [^3^H]5-HT/[^3^H]NA efflux (S1, S2) was expressed as the area-under-the-curve of the net release. Previous HPLC analyses showed that tritium efflux is a good marker of [^3^H]5-HT/[^3^H]NA release under similar experimental conditions [43], [52].

### ^3^H-Citalopram, ^3^H-Nisoxetine and ^3^H-Dihydroalprenolol Binding Experiments

Male mice (80–85 days old) were decapitated, and the hippocampus was dissected, weighted, and frozen overnight at −70°C. The next day, the tissue from four animals was pooled, homogenized in incubation buffer using an Ultra-Turrax and centrifuged at 40 000×*g* for 15 minutes. The pellet was washed by repeated resuspension in buffer, followed by centrifugation. The final pellet was resuspended in 30 volumes (w/v) of buffer and used immediately for the binding experiments. ^3^H-Citalopram (sp.act. 70 Ci/mmol) and ^3^H-Nisoxetine (sp.act. 85.5 Ci/mmol) binding was performed in a final volume of 250 µl of 50 mM Tris-HCl buffer, pH 7.4, containing 120 mM NaCl and 5 mM KCl. ^3^H-DHA (sp.act. 88 Ci/mmol) binding was measured in 50 mM Tris-HCl buffer, pH 7.4. Eight ligand concentrations (0.1–4 nM) were used in three parallels. The incubation time was 1 hour at 25°C for citalopram, 2.5 hrs at 4°C for nisoxetine and 30 minutes at 25°C for dihydroalprenolol. The non-specific binding was determined in the presence of 1 µM paroxetine (citalopram), 10 µM desmethylimipramine (nisoxetine) and 1 µM (-) alprenolol (DHA). The membranes of P2rx7+/+ and P2rx7−/− mice were assayed in parallel. The binding was terminated using vacuum filtration on GF/B filters soaked in 0.05% polyethylenimine. The K_d_ and the number of binding sites (B_max_) were calculated using Prism3 software. The protein concentration (approx. 0.5 mg/ml) was measured according to Lowry method [50] using CuEDTA.

### Measurement of^3^H-5HT Uptake in Synaptosomal (P2) Preparation of Mouse Hippocampus

The hippocampus of each mouse (80–85 days old) was homogenized in 0.32 M sucrose solution (1∶10 g/ml) using a Teflon pestle homogenizer and centrifuged at 1,000×*g* for 10 minutes. The supernatant was centrifuged at 12,000×*g* for 20 minutes. The pellet containing synaptosomes was suspended in 0.32 M sucrose (1∶30 g/ml). Aliquots of the synaptosomal suspension were preincubated for 5 min at 37**°**C, in a final volume of 1 ml oxygenated (95% O_2_+5% CO_2_) Krebs solution containing 118 mM NaCl, 4.7 mM KCl, 2.5 mM CaCl_2,_ 1.2 mM KH_2_PO_4_, 1.2 mM MgSO_4_, 10 mM D-glucose, 25 mM NaHCO_3_, 0.3 mM ascorbic acid and 0.01 mM pargyline (pH 7.4). After preincubation, ^3^H-5HT (10 nM) was added, and the incubation was continued for 10 min. The uptake was terminated by addition of cold physiological saline, and by vacuum filtration of the samples through GF/B filters. The non-specific uptake was determined by incubation of the samples at 0°C. The protein content was measured using Lowry method [50]. The uptake in synaptosomal preparations from P2rx7+/+ and P2rx7−/− mice was measured in the same assay.

### Materials

3′-O-(4-benzoyl-benzoyl)adenosine 5′-triphosphate, (BzATP, Tocris), bacterial lipopolysaccharide (LPS, serotype 055:B5, Sigma), Brilliant blue G, (BBG, Tocris), 6-cyano-7-nitroquinoxaline-2,3-dione (CNQX, Tocris), 4,4′,4′′,4′′′-[Carbonylbis(imino-5,1,3-benzenetriyl-*bis*(carbonylimino))]*tetrakis*-1,3-benzenedisulfonic acid, octasodium salt (NF449, Tocris), 3,5-dihydroxyphenylglycine, (DHPG, Tocris), α-Methyl-4-carboxyphenylglycine (MCPG, Tocris), (α*R*,β*S*)-α-(4-Hydroxyphenyl)-β-methyl-4-(phenylmethyl)-1-piperidinepropanol maleate (RO-256981, Tocris), 3-Chloro-4-fluoro-N-[4[[2(phenylcarbonyl)hydrazino]carbonyl]benzyl] benzenesulfonamide (TCN-201, Tocris), [^3^H]glutamic acid ([^3^H]Glu), [^3^H]-5HT, [^3^H]-NA, [^3^H]-Citalopram, [^3^H]-Nisoxetine, [^3^H]-Dihydroalprenolol (Perkin Elmer), sucrose (Sigma).

### Statistics

All data were presented as the mean ± SEM of *n* determinations. The statistical analyses were performed using one-way ANOVA followed by Dunnett’s post-hoc test (multiple comparisons) and the unpaired Student’s t-test (pair-wise comparisons), as appropriate. The SPT datasets and resting [^3^H]Glu outflow were analyzed by two-way ANOVA followed by Fischer LSD test. The level of significance was set at P<0.05.

## Results

### Behavior Experiments

**Figure 1:**
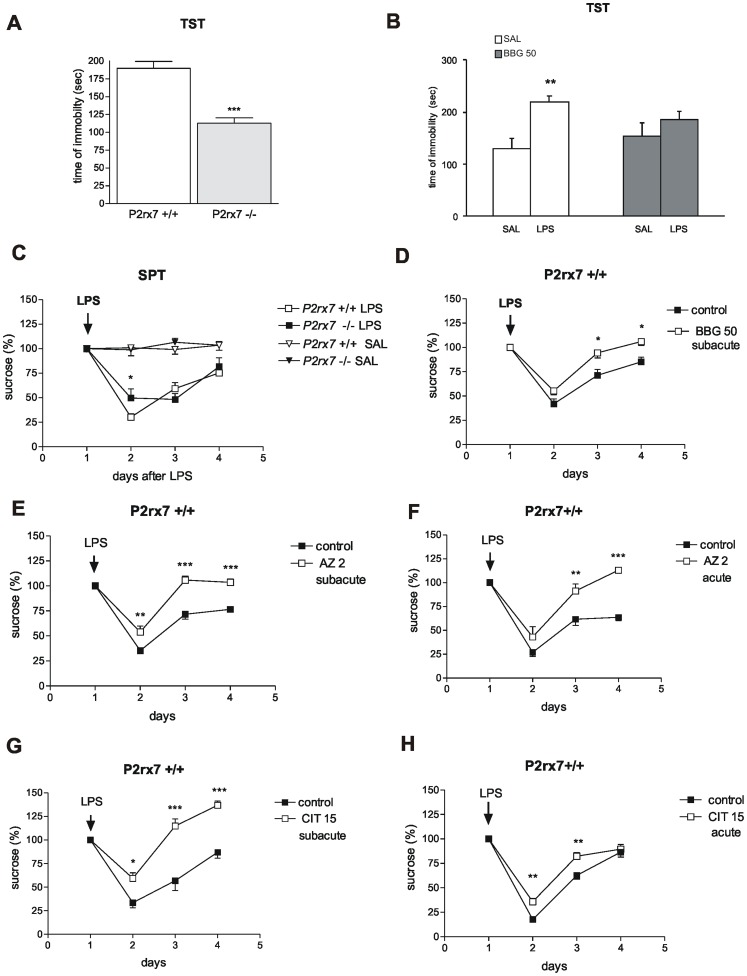
Genetic deletion and pharmacological inhibition of P2rx7 results in antidepressant phenotype in mice using the TST (A, B) and SPT (C–H) tests. A/Genetic disruption of P2rx7 expression decreases basal immobility in the TST (n = 6,10, ***P<0.001 vs. P2rx7+/+, Student *t* test). The immobility time is expressed in sec. The total test period was 360 sec. B/Effect of acute BBG treatment (50 mg/kg i.p.) on LPS-induced depressive behavior in TST. LPS was added at a dose of 1 mg/kg at 24 h before testing (n = 7–9, **P<0.01 vs. SAL, Student *t* test). BBG was administered intraperitoneally at 30 min before testing; saline-treated mice (SAL) were injected with equal volume of saline. C/LPS-induced decline in sucrose preference is attenuated in P2rx7−/− mice. Baseline sucrose intake was measured under a habituation period of 3–4 days before treatment, and the mean volume ingested over the last 3 training sessions was used as a baseline value. The mice were treated with LPS (0.2 mg/kg i.p.) or an equal volume of saline (SAL), indicated by an arrow, and the sucrose intake was monitored over subsequent days. LPS substantially decreased sucrose consumption as a sign of anhedonia (F_LPS_(1, 63) = 1193; P<0.0001). The sucrose consumption was expressed as a percentage of the baseline (%). N = 9–14, *P<0.05 vs. P2rx7+/+. Two-way ANOVA, followed by Fischer LSD test. D-H/The P2X7 receptor antagonists Brilliant blue G (BBG), AZ-10606120 (AZ), and the selective 5-HT re-uptake inhibitor citalopram (CIT) inhibit the development of LPS-induced anhedonia in P2rx7+/+ mice. D/The antidepressant effect of a subacute, 4-day treatment with BBG (50 mg/kg i.p./day, n = 8–20, *P<0.05, vs. control). E/The effect of subacute (AZ 2 subacute) AZ-10606120 treatment on LPS-induced anhedonia. Notably, in this protocol, AZ-10606120 significantly attenuated the decline in sucrose consumption at 24 h after LPS treatment. (F_AZ_ (1,39) = 1179.4, P<0.00001; n = 5–9, **P<0.01, ***P<0.001 vs. control). F/The effect of acute AZ-10606120 (AZ 2, acute) treatment on LPS-induced anhedonia. When mice were pretreated with AZ-10606120 for 30 min before LPS injection at the doses indicated in the legend (mg/kg), sucrose consumption on the 2^nd^ and 3^rd^ day after LPS injection was significantly higher (n = 7–8, **P<0.01, ***P<0.001 vs. control). G and H/. Effect of 4-day subacute (CIT 15 subacute, G), and single acute treatment with citalopram (CIT 15, acute, H) on LPS-induced anhedonia. Citalopram elicited a comparable effect to AZ-10606120 using both application protocols (n = 5–10, **P<0.01, ***P<0.001 vs. control). The sucrose consumption in all experiments was evaluated at 0, 24, 48 and 72 h after LPS injection and expressed as a percentage of the baseline. Two-way ANOVA, followed by Fischer LSD test was performed as statistical analyses in SPT datasets. 2–3 months old drug- and test-naive male homozygous mice (P2rx7+/+ and P2rx7−/−), weighing approximately 30 g were used in the experiments.

Using automated recording, the basal time of immobility was 189.7±9.7 sec (n = 6) during the 6-min tail suspension test (TST) in P2rx7+/+ mice. This value significantly decreased in the P2rx7−/− mice (Fig. 1A). The time of immobility was also examined 24 h after LPS (1 mg/kg i.p.) injections in P2rx7+/+ mice, i.e., when acute symptoms of the LPS induced sickness behavior is restored [41]. The basal time of immobility was significantly increased in response to systemic LPS challenge, i.e., the mice displayed depressive-like behavior (Fig. 1B). The LPS-induced depressive behavior was significantly attenuated in mice pretreated with the selective P2rx7 antagonist, Brilliant blue G (50 mg/kg i.p.) (Fig. 1B).

In addition to TST, which examines active coping with behavioral stress, we also employed the sucrose preference test (SPT) to assess anhedonia, which is another core symptom of depression [53]. The basal sucrose consumption in P2rx7+/+ mice was 25.04±0.76 g/100 g (n = 74), and their water and food consumption was 7.1±0.32 g/100 g and 10.57±0.27 g/100 g (n = 74), respectively. In these experiments, a lower (200 µg/kg i.p.) LPS dose was applied, which elicited a relatively selective anhedonia response in our preliminary experiments. Indeed, the LPS (200 µg/kg i.p.) injection elicited a significant decrease in sucrose, but not water consumption at 24 h later in P2rx7+/+ mice (Fig. 1C), indicating the development of anhedonia; the sucrose consumption was restored in the subsequent days and approached the baseline value at 3 days after the treatment (Fig. 1C). The basal sucrose consumption in P2rx7−/− mice was 25.89±0.94 g/100 g (n = 60), which was not significantly different from that observed in P2rx7+/+ mice. The water consumption was 6.58±0.29 g/100 g (n = 60, P>0.05), and the food consumption was 9.82±0.25 g/100 g (n = 60, P>0.05), which was also similar to those observed in P2rx7+/+ mice. The LPS treatment also decreased sucrose consumption in P2rx7−/− mice; however, the decrease was less pronounced (Fig. 1C).

Next, we examined the effect of two P2rx7 antagonists, BBG and AZ-10606120, in comparison with the selective 5-HT re-uptake inhibitor antidepressant drug, citalopram, which was used as a reference compound. A subacute, 4-day application of BBG (50 mg/kg i.p.) did not change the sucrose consumption in P2rx7+/+ mice at one day after LPS injection, but this treatment significantly increased sucrose consumption over subsequent days (Fig. 1D), i.e., the mice exhibited an antidepressant phenotype. The administration of AZ-10606120 (2 mg/kg i.p.), another P2rx7 antagonist, elicited a more robust antidepressant response, which not only resulted in the restoration of the decline in sucrose consumption, but the amount of sucrose consumed on the day after LPS injection was significantly higher after the subacute treatment (Fig. 1E). Treatment with AZ-10606120 (2 mg/kg i.p.) also promoted the restoration of the anhedonia upon acute injection, and on the 3^rd^ day after LPS injection, the mice consumed significantly higher amount of sucrose (Fig. 1F). Similar responses were observed after 4-day subacute (Fig. 1G) and acute (Fig. 1H) treatments with citalopram. None of the treatments significantly altered water consumption under basal conditions or after LPS treatments (data not shown).

**Figure 2:**
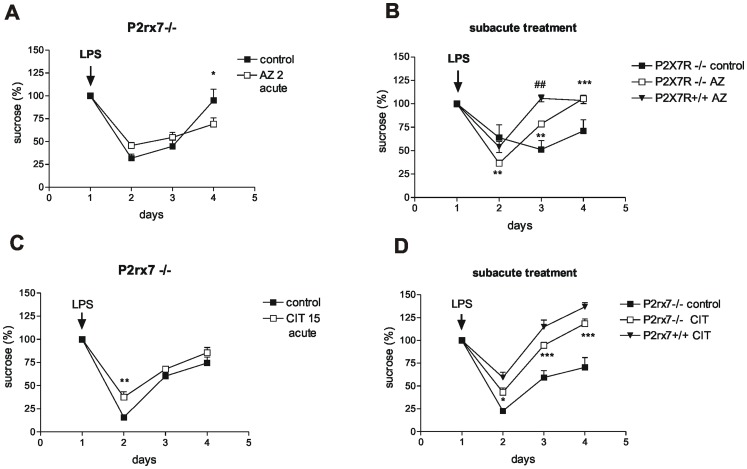
The effect of P2X7 receptor antagonist AZ-10606120 (AZ) and citalopram (CIT) on LPS-induced anhedonia in P2rx7−/− mice. A and C/The effect of acute AZ-10606120 (AZ 2 acute, A) and citalopram (CIT 15 acute, C) treatment on LPS-induced anhedonia. The protocols were identical to those depicted in Fig. 1 F and H, respectively. The inhibitory effect of AZ-10606120, but not citalopram, was abolished in P2rx7−/− mice (n = 4–11, *P<0.05, **P<0.01 vs. control). B/D/Effect of subacute, 4-day treatment with AZ-10606120 (AZ, B) and citalopram (CIT, D) on LPS-induced anhedonia. The protocol was identical to the experiments depicted in Fig. 1 E and G, respectively. Although both citalopram and AZ-10606120 alleviated anhedonia over subsequent days in these mice, the effect of AZ-10606120, but not citalopram, was significantly attenuated compared with the effect measured in P2rx7+/+ mice (n = 5–11, **P<0.01, ***P<0.001 vs. control, ^##^P<0.01 vs. subacute AZ in P2rx7+/+ mice, two-way ANOVA, followed by Fischer LSD test).

The inhibitory effect of acute AZ-10606120 (2 mg/kg i.p.) treatment on LPS-induced anhedonia was completely absent in P2rx7−/− mice (Fig. 2A). The effect of subacute AZ-10606120 (2 mg/kg i.p.) treatment on LPS-induced anhedonia was also significantly attenuated in the deficiency of P2rx7 (Fig. 2B). By contrast, the effect of acute (Fig. 2C) and subacute (Fig. 2D) citalopram treatment was largely preserved in P2rx7−/− mice.

### [^3^H]Glutamate Release in Acute Hippocampal Slices

**Figure 3:**
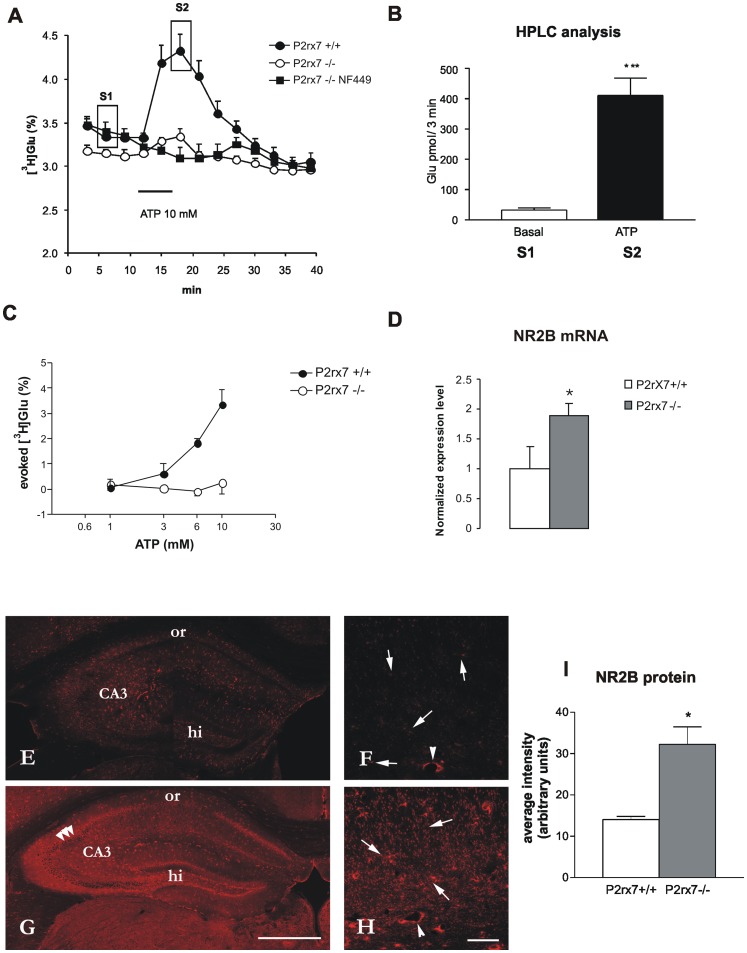
The effect of the genetic deletion of P2rx7 on ATP-evoked tritiated (A, C) and endogenous (B) glutamate efflux (D) mRNA expression and (E–I) immunofluorescence staining of the NR2B subunit of the NMDA receptors in acute hippocampal slices. A/10 mM ATP was used to induce [^3^H]Glu release from hippocampal slices of P2rx7+/+ and P2rx7−/− mice. After a 60-min preperfusion, the basal extracellular [^3^H]Glu efflux was lower in P2rx7−/− mice. 6-min perfusion of ATP (10 mM) resulted in a transient increase in the efflux of [^3^H]Glu in P2rx7+/+ mice, which peaked at 6 min after ATP administration and gradually decreased to baseline levels after 12 min. The ATP-evoked [^3^H]Glu efflux is substantially decreased in the hippocampus of P2rx7−/− mice, and the residual efflux is abolished by the selective P2X1 receptor antagonist NF449 (100 nM), which was applied at 15 min before ATP perfusion. [^3^H]Glu release is expressed as a percentage of the amount of radioactivity in the tissue at the sample collection time (fractional release). For the evaluation of the basal tritium outflow, the tritium content of the first four consecutive 3-min samples were taken into account. The curves represent the mean ± SEM of 8–12 identical experiments. B/HPLC analysis. Samples indicated in (A) as S1 and S2 were analyzed. ATP perfusion (10 mM) significantly increases the endogenous Glu level in the effluent. Results are expressed as pmol/3 min. N = 8, ***P<0.001. C/Concentration-response relationship of ATP-evoked [^3^H]Glu efflux in P2rx7+/+ and P2rx7−/− mice. Experiments were performed according to the protocol shown in (A), using different concentrations of ATP, as indicated in the abscissa. The net ATP-induced release was calculated and expressed as the fractional release (%). The curves represent the mean ± S.E.M. of 4–12 identical experiments. D/Changes in the mRNA expression levels of the NMDA-NR2B receptor in hippocampus obtained from P2rx7+/+ and P2rx7−/− mice. Immediately after the 60-min incubation, the brain slices were removed and total RNA was extracted from the hippocampus and reverse transcribed to cDNA. Quantitative SYBR Green real-time PCR was performed using specific primers, as described in Methods, and cDNA as a template. The experiments were repeated two times with similar results. The expression level of the NR2B receptor was normalized to that of the distinct housekeeping gene, 18S rRNA. The data are displayed as the mean ± SEM. Asterisk indicates significant difference from the P2rx7+/+ mice (*P<0.05, Student’s t-test). E-I/Immunofluorescence staining for NR2B on hippocampal sections of P2rx7+/+ and P2rx7−/− mice. Besides the typical dotted staining most likely presenting NR2B - immunolabelled terminals (arrows, F, H) some staining is observable around cell bodies especially in the pyramidal cell layer (three arrowheads G). The whole staining is more intense in the hippocampal section of P2rx7−/− mouse (G, I). The most intense staining is observed in the CA3 regions (CA3 on E, G and F, H), while stratum oriens (or) shows the least immunoreactivity either in P2rx7+/+ and P2rx7−/− sample. Contrary to P2rx7+/+ staining (E), intense immunofluorescence illustrates hilus region on P2rx7−/− (G) sample. Images acquired at higher magnification (F,H) also show blood vessels (arrowheads), stained at the same level in both sections (background staining). bars: 50 µm in F, H, 500 µm in E, G. I. Immunofluorescence staining intensity for NR2B in P2rx7+/+ and P2rx7−/− mice. Average intensity was quantified with NIH ImageJ program (U.S. National Institutes of Health, Bethesda, MD) and is expressed in arbitrary units. Asterisk indicates significant difference from the wild type mice (*P<0.05, Student’s t-test, n = 3).

After loading the hippocampal slices with [^3^H]Glu, the uptake of radioactivity was 380±68 kBq/g (n = 12) in the hippocampal slices of P2rx7+/+ mice and 390±46 kBq/g (n = 8, P>0.05) in the P2rx7−/− mice; these results were not significantly different. The basal efflux of [^3^H]Glu, when measured in a single sample was 3.33±0.08% (n = 12) and 3.14±0.05% (n = 8, P>0.05) in P2rx7+/+ and P2rx7−/− mice, respectively. However, when four consecutive samples under drug free conditions were taken into account, two-way ANOVA indicated a significant genotype effect (F(1,27), P<0.001, Fig. 3A), which is indicative for a lowered extracellular Glu level in the hippocampus of P2rx7 deficient mice. A 6-min perfusion with the P2rx7 agonist, ATP (1–10 mM), elicited a rapid and concentration-dependent increase in the efflux of [^3^H]Glu, which was reversible upon washout (Fig. 3A, C). The net release of tritium evoked in response to 10 mM ATP was 3.34±0.57% (n = 12, Fig. 3C). The endogenous glutamate content of the samples collected during resting condition and at the peak of ATP-evoked response was also determined using HPLC analysis (Fig. 3B), and a remarkable elevation in the level of glutamate was observed in response to ATP application.

When the hippocampal slices derived from P2rx7−/− mice were challenged with ATP, using an identical protocol, only a slight elevation in the efflux of [^3^H]Glu was detected at ATP concentrations of 10 mM (0.25±0.43%, n = 8, P<0.001, Fig. 3A) and lower (Fig. 3C). The residual elevation of [^3^H]Glu efflux in the presence of ATP (10 mM) was sensitive to inhibition through the P2X1 receptor selective antagonist NF449 (100 nM) (Fig. 3A). In contrast, the selective P2rx7 antagonist Brilliant blue G (100 nM) did not affect the residual [^3^H]Glu efflux in P2rx7−/− mice (0.56±0.25%, n = 9, P>0.05).

### Analysis of NR2B mRNA Expression Using Quantitative Real-time PCR and NR2B Protein Immunofluorescence

Hippocampal slices from P2rx7+/+ and P2rx7−/− mice were incubated *in vitro* with Hibernate medium containing Krebs solution for 60 min. Changes in the level of mRNA transcripts of the NR2B glutamate receptor subunits were measured using real-time RT-PCR. The gene expression level was normalized to the expression of the 18S rRNA reference gene. The results revealed that the NR2B receptor mRNA level was upregulated to 1.89±0.0015 of the corresponding wild type values, (established as 1) normalized to 18S rRNA (Fig. 3D, n = 4, P<0.05).

The NR2B protein immunostaining was not evenly distributed on the hippocampal sections of P2rx7+/+ and P2rx7−/− mice (Fig. 3E, F, G, H). In general, the staining was more intense on sections of P2rx7−/− origin (Fig. 3I). Whereas high immunoreactivity for N2RB was found in hilus region in hippocampal sections of P2rx7−/− mice and weak in the P2rx7+/+ mice, the staining pattern was otherwise rather similar in both cases (Fig. 3 E, G). The most intensive punctate immunostainng was observed in the CA3 region, while stratum oriens was weakly stained.

### BDNF Protein Assay

**Figure 4:**
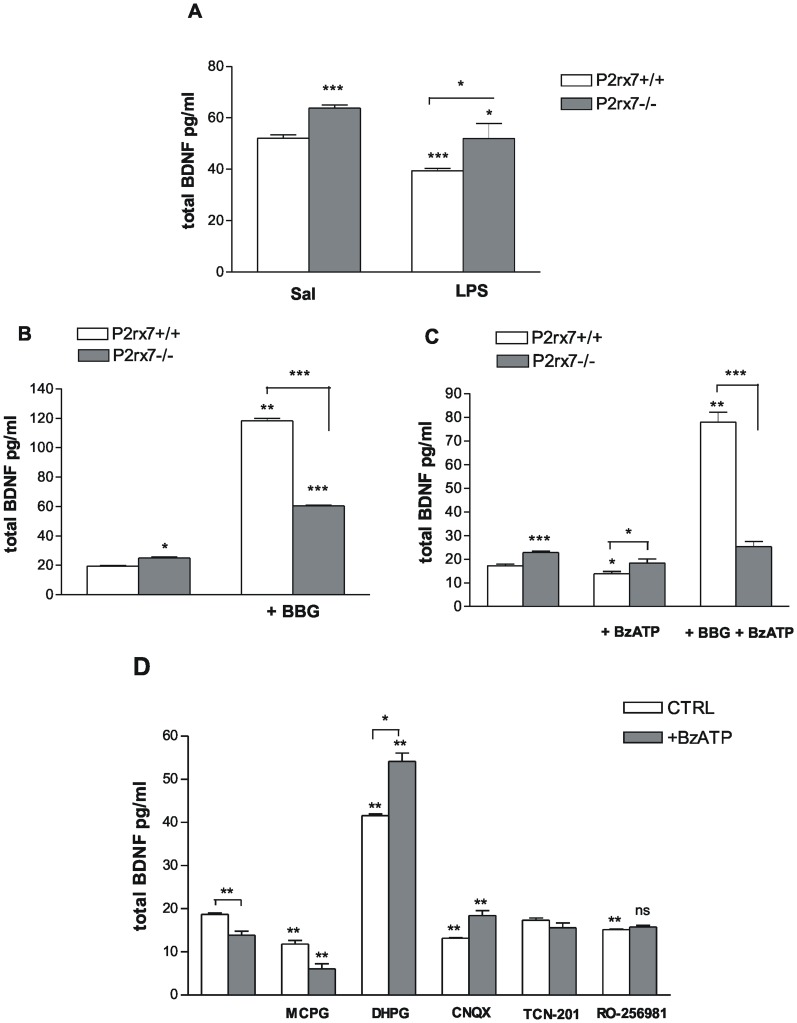
P2rx7 participates in the regulation of the basal BDNF expression in the mouse hippocampus. A/BDNF protein expression in the hippocampus of P2rx7+/+ and P2rx7−/− mice after saline (Sal) and LPS (1 mg/kg i.p.) treatment. Intraperitoneal injection of LPS significantly decreased the level of BDNF expression in the hippocampus of P2rx7+/+ mice at 24 h after treatment compared with saline treatment (Sal). The BDNF protein level was significantly higher in the hippocampus of saline treated P2rx7−/− mice. The animals were sacrificed at 24 h after the injection of LPS/saline. The data are given as the mean level of neurotrophin ± SEM. Asterisks indicate significant differences between the saline and LPS-treated groups and between the WT and P2rx7−/− groups (n = 4–6, *P<0.05, *** P<0.001). B, C Effect of the P2X7 receptor antagonist BBG (B) and the P2X agonist BzATP (C) on the basal level of BDNF in the hippocampus of P2rx7+/+ and P2rx7−/− mice. The P2rx7 antagonist Brilliant Blue G (BBG, 100 nM) was applied 15 min before the start of BzATP incubation, and BzATP (100 µM) was added for 30 min. Notably, in the experiments with BzATP (C), Mg^2+^ was omitted from the incubation solution (see Methods). The data are given as the mean level of neurotrophin ± SEM. Asterisks indicate significant differences calculated by one-way ANOVA followed by Dunnett’s test (multiple comparisons) and Student’s t-test (pairwise comparisons) (*P<0.05, **P<0.01,***P<0.001). D/Effect of glutamate receptor antagonists and agonist on the basal and BzATP-induced decrease in BDNF expression in hippocampal slices of P2rx7+/+ mice. The inhibitory effect of BzATP (100 µM) could be counteracted using CNQX (10 µM), the non-NMDA-type glutamate receptor antagonist, TCN-201 (10 µM), the NMDA-NR1/NR2A glutamate receptor antagonist and RO-256981 (3 µM) the NMDA-NR2B glutamate receptor antagonist but not using MCPG (200 µM), the group I mGluR antagonist. CNQX, TCN-201 and RO-256981 was added for 15 min, MCPG was applied for 30 min, and the group I mGluR agonist, DHPG (100 µM), was administered for 10 min. Data are given as the mean level of neurotrophin ± SEM with and without (first columns) the indicated antagonists in the presence (BzATP) or absence (CTRL) of BzATP. Asterisks indicate significant differences calculated using one-way ANOVA followed by Dunnett’s test and Student’s t-test (ns P>0.05, **P<0.01), as appropriate, indicated by the horizontal bars.

In subsequent experiments, P2rx7+/+ and P2rx7−/− mice were challenged with an i.p. injection of 1 mg/kg LPS, and the BDNF expression was evaluated in the hippocampus at 24 hours after the treatment. The basal level of BDNF in the hippocampus of P2rx7+/+ mice at 24 h after saline administration was 52.04±1.31 pg/ml (Fig. 4A, n = 6). The systemic LPS administration caused a remarkable decrease in BDNF levels (Fig. 4A, 39.46±0.85 pg/ml, n = 8, P<0.001; 24.11% decrease). The basal BDNF level in the hippocampus of P2rx7−/− mice was 63.8±1.15 pg/ml (n = 8), which was significantly higher than in P2rx7+/+ mice (Fig. 4A, P<0.001). However, the LPS treatment also attenuated the BDNF protein expression in the hippocampus of P2rx7−/− mice (P2rx7−/−: 51.97±5.87 pg/ml, 17.46% decrease; n = 8, P<0.05, Fig. 4A).

To explore the regulatory role of P2X7 receptors in the local modulation of BDNF production, the neurotrophin levels in hippocampal slices in P2rx7+/+ and P2rx7−/− mice were subsequently analyzed *in vitro*. In these experiments, hippocampal slices were incubated in the presence of agonist and antagonist of P2rx7 and their effects on BDNF production were examined. Consistent with the *ex vivo* results, the basal BDNF level in the hippocampal slices of P2rx7−/− mice in these experiments was significantly higher than detected in P2rx7+/+ mice (Fig. 4B, C, 23.94±0.49 pg/ml and 18.32±0.39 pg/ml in P2rx7−/− and P2rx7+/+ mice, n = 8/group, P<0.001).

The selective P2rx7 antagonist, Brilliant Blue G (BBG; 100 nM), significantly enhanced the basal level of BDNF in the presence of the P2X7 receptor (Fig. 4B; 118.22±1.59 pg/ml, 656% increase; n = 4, P<0.01). Therefore, the pharmacological inhibition of P2X7 receptors reproduced the effect of genetic deletion in P2rx7+/+ mice. Although BBG also increased the BDNF protein level in the hippocampus of P2rx7−/− mice (P2rx7−/−: 60.31±0.31 pg/ml, 272.7% increase; n = 4, P<0.001), its stimulatory effect was significantly attenuated in the deficiency of the P2X7 receptor (Fig. 4B).

Next, we evaluated the effect of the P2X receptor agonist 3′-O-(4-benzoyl-benzoyl) adenosine 5′-triphosphate (BzATP) on the hippocampal BDNF protein level. Because previous studies have shown that P2rx7 agonist–induced responses are amplified in Mg^2+^-free medium e.g. [18], in the subsequent experiments, Mg^2+^ was omitted from the BzATP incubation medium. BzATP (100 µM) caused a decrease in the basal level of BDNF production in P2rx7+/+ mice (Fig. 4C; P2rx7+/+: 13.85±0.98 pg/ml, 25.73% decrease; n = 8, P<0.05) without significantly affecting the BDNF level in the P2rx7−/− mice (Fig. 4C). Moreover, the observed inhibitory effect of BzATP was reversed by BBG (Fig. 4C; BBG+BzATP: 77.87±4.24 pg/ml, n = 4), indicating that this inhibitory action is due to the activation of P2rx7.

Literature data and the previous experiments suggested that the activation of P2rx7 by ATP or BzATP elicits Ca^2+^ influx [18], which is followed by an increased glutamate release [19], [20], [21], [22]. Therefore, we examined whether the P2rx7-mediated glutamate release and its action on various glutamate receptors might be responsible for P2rx7-mediated alterations in BDNF levels. To this end, the effect of BzATP on the basal BDNF level was examined in the presence of the Group I (mGluR_1,5_) mGluR antagonist, MCPG (200 µM), CNQX (10 µM) the non-NMDA-type glutamate receptor antagonist, TCN-201 (10 µM) the NR1/NR2A glutamate receptor selective antagonist and the NR2B glutamate receptor antagonist, RO-256981 (3 µM). Among the antagonists, the inhibitory effect of BzATP (100 µM) was occluded by CNQX, TCN-201 and by RO-256981 (Fig. 4D; RO-256981 alone: 15.20±0.12 pg/ml, n = 4; BzATP+RO-256981∶15.75±0.44 pg/ml, n = 12, P>0.05) indicating the involvement of both NMDA and non-NMDA-type ionotropic glutamate receptors in this effect. In contrast, blockade of mGluR_1,5_ receptors using MCPG did not antagonize the inhibitory effect of BzATP (Fig. 4D, BzATP+MCPG: 6.09±1.17 pg/ml, 67.34% decrease n = 4, P<0.01). Moreover, when applied alone, MCPG significantly diminished the basal level of hippocampal BDNF, suggesting that the endogenous activation of Group I mGluR contributes to BDNF production in the hippocampus. The Group I mGluR agonist, DHPG, caused a profound elevation in the basal level of BDNF production in P2rx7+/+ mice (Fig. 4D; DHPG: 41.59±0.40 pg/ml, n = 4, P<0.01). Interestingly, BzATP significantly increased BDNF levels in the presence of DHPG (Fig. 4D; BzATP+DHPG: 54.14±1.92 pg/ml, n = 4, P<0.05).

### BrdU Incorporation Experiments

**Figure 5:**
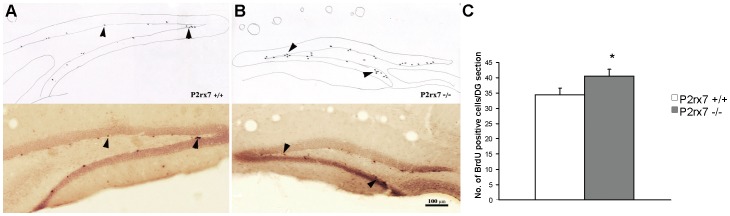
Summary of BrdU staining in P2rx7+/+ (A) and P2rx7−/− (B) mice. A, B/Representative sections show rostral hippocampal DG areas in 1×1 sections of male wild type and P2rx7 knock out mice. Dark dots (*arrowheads*) represent the BrdU-positive cells (ImmPress-DAB-Ni staining). Camera lucida drawings of the same sections where the newly formed BrdU-labeled cells are indicated. The microscopic picture and drawing were taken at the same magnification (20X), and the bar indicates 100 µm. Histogram showing the average number of BrdU-positive cells in a rostral hippocampal DG area in the granule cell layer and in the 50-µm zone adjacent to its inner edge. C/We observed a significant difference (n = 5, p = 0.046) in the average number of labeled cells in the sections of P2rx7+/+ and P2rx7−/− mice.

In these experiments, we used the proliferation marker BrdU to evaluate adult neurogenesis and examined the average number of BrdU-positive cells in the dentate gyrus (DG), which contains the granular cell layer and its subgranular zone at no more than 50 µm apart. There was a significantly higher average number of BrdU-positive cells in the DG of the rostral hippocampal sections of P2rx7−/− mice compared with P2rx7+/+ mice (Fig. 5A, B, C). Although the difference between the two genotypes was not robust, we consistently observed, except in one case, more BrdU-positive cells in the DG of P2rx7−/− mice than in their P2rx7+/+ littermates.

### Dysregulation of Biogenic Amine Levels in the Hippocampus of P2rx7−/− Mice

**Figure 6:**
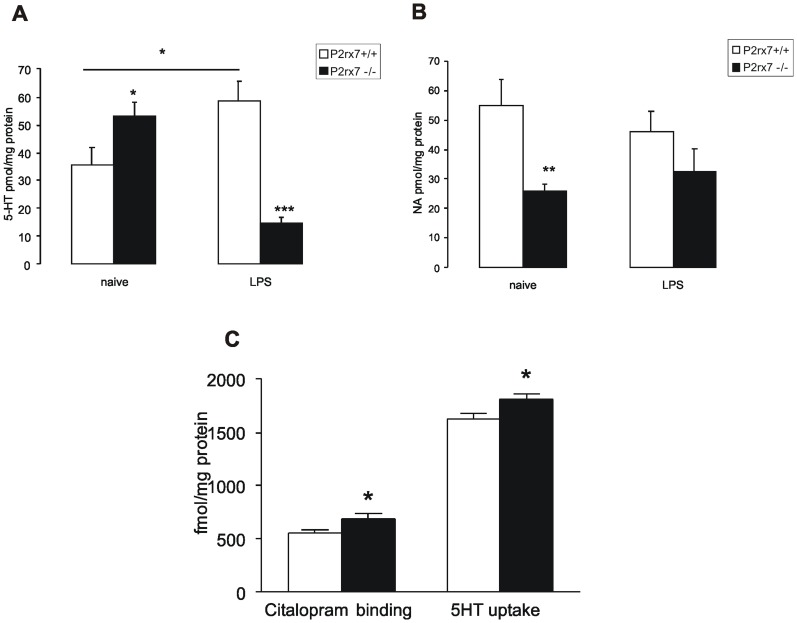
Genetic deletion of P2rx7 leads to the alteration of NA and 5-HT levels (A, B), elevation of^3^H-Citalopram binding sites and 5-HT uptake (C) in the mouse hippocampus. A, B/NA and 5-HT levels were analyzed using HPLC in the hippocampus of naive and LPS (1 mg/kg)-treated P2rx7+/+ and P2rx7−/− mice. A/5-HT levels are significantly elevated in the hippocampus of untreated P2rx7−/− mice. LPS treatment increases 5-HT levels in the hippocampus of P2rx7+/+ mice. This elevation is absent in P2rx7−/− mice. B/NA levels are significantly decreased in the hippocampus of untreated, but not in LPS-treated P2rx7−/− mice. NA and 5-HT levels are expressed as pmol/mg protein. Data are given as the mean ± SEM of 13–22 experiments. Asterisks denote significant differences related to genotype or LPS treatment (*P<0.05, **P<0.01, ***P<0.001). C/The number (B_max_) of [^3^H]Citalopram recognition sites was increased in the membrane preparation, and the synaptosomal 5-HT uptake was enhanced in the hippocampus of P2rx7−/− mice (black bars) compared with their P2rx7+/+ littermates (clear bars). Citalopram binding was expressed as fmol/mg protein, and the 5-HT uptake was expressed as fmol/mg protein/10 minutes. Experiments in P2rx7+/+ and P2rx7−/− mice were performed in the same assay. Binding parameters were calculated using Prism3 software. For experimental procedures, see Methods section. Data are given as the mean ± SEM of four independent experiments, * P<0.05.

The 5-HT and NA content was measured using HPLC in the hippocampus of naïve, untreated and LPS-treated (1 mg/kg i.p.) P2rx7+/+ and P2rx7−/− mice. A significantly elevated basal expression of 5-HT was observed in untreated P2rx7−/− mice (Fig. 6A) compared with P2rx7+/+ mice, whereas the 5HIAA/5-HT ratio was profoundly decreased in the hippocampus of P2rx7−/− mice (2.07±0.57 and 0.31±0.08 in P2rx7+/+ and P2rx7−/− animals, respectively, n = 14–22, P<0.01). The NA levels were also significantly decreased in response to genetic deletion (Fig. 6B).

LPS treatment significantly increased 5-HT levels in the hippocampus (Fig. 6A) of P2rx7+/+ mice. This elevation was absent in P2rx7−/− mice; moreover, the 5-HT level was decreased in response to endotoxin treatment (Fig. 6A). By contrast, LPS treatment did not affect the NA content in the hippocampus in either genotype (Fig. 6B).

### [^3^H]5-HT/[^3^H]NA Release Experiments

As a functional readout of monoaminergic neurotransmission [^3^H]5-HT and [^3^H]NA release experiments were also performed in acute hippocampal slices.

In [^3^H]5-HT release experiments, there was no significant difference in the resting- and electrically-evoked [^3^H]5-HT efflux between P2rx7+/+ and P2rx7−/− mice (Table 1). Likewise, when tissue slices were loaded with [^3^H]NA, the resting [^3^H]NA release was similar in the hippocampal slices of P2rx7+/+ and P2rx7−/− mice (Table 1). As a potential correlate of decreased NA levels in the deficiency of functional P2rx7, the electrical stimulation evoked [^3^H]NA efflux in the hippocampus was slightly, but significantly decreased in P2rx7−/− mice (Table 1).

**Table 1:** Resting and electrical stimulation evoked[^3^H]5-HT and [^3^H]NA efflux in the hippocampus of P2rx7+/+ and P2rx7−/− mice, respectively.

	[^3^H]5-HT		[^3^H]NA	
	P2rx7+/+	P2rx7−/−	P2rx7+/+	P2rx7−/−
Resting efflux (Bq/gx10^3^)	3.57±0.29 (12)	3.28±0.18 (12)	3.40±0.88 (12)	2.41±0.21 (12)
Evoked efflux (S1, Bq/gx10^3^)	4.12±0.7 (12)	3.68±0.66 (12)	14.61±2.29 (12)	6.73±2.08* (12)

Tissue slices were loaded with [^3^H]5-HT or [^3^H]NA and then superfused with Krebs’ solution. After 60 min preperfusion, slices were stimulated electrically with the following parameters: 25 V, 1 msec, 2 Hz, 240 shocks. The release of radioactivity was expressed in Bq/g. For the calculation of the resting [^3^H]5-HT/[^3^H]NA efflux, the tritium content of the sample collected immediately before the first electrical stimulation period was taken into account. Electrical stimulation-induced [^3^H]5-HT/[^3^H]NA efflux (S1) was expressed by calculating the net release in response to electrical stimulation by the area-under-the-curve method. The number of experiments are in parentheses. *P<0.05, significantly different from P2rx7+/+ mice, calculated by the Student’s t-test.

### ^3^H-5HT Uptake and ^3^H-Citalopram Binding are Enhanced in the Hippocampus of P2rx7 Knockout Mice

To examine, whether alterations in tissue monoamine levels results from a change in the uptake, we examined the 5-HT and NA transporters using the specific ligands, [^3^H]Citalopram and [^3^H]Nisoxetine, and measured the 5-HT uptake in synaptosomal preparations. As shown in Fig. 6C, the uptake in the synaptosomal preparation of the P2rx7+/+ mouse hippocampus was 1627±47 fmol/mg protein (n = 4) using 5×10^−8^ M 5-HT, whereas the uptake slightly but significantly (P<0.04) enhanced to 1816±52 fmol/mg protein (n = 4) in P2rx7−/− mice. The [^3^H]Citalopram-labeled 5-HT transporter demonstrated saturable binding with no significant changes in the K_d_ value in the hippocampal membranes of either genotype (Table 2); however, a significant increase was observed in the B_max_ of P2rx7−/− mice compared with P2rx7+/+ littermates (Fig. 6C, Table 2). To assess, whether the deletion of P2rx7 affected the NA transporter, the binding of the specific ligand ^3^H-Nisoxetine was examined. No difference was observed in ^3^H-Nisoxetine binding parameters between P2rx7+/+ and P2rx7−/− mice in the hippocampus (Table 2). As β-adrenergic receptors are downregulated after chronic antidepressant treatment [54], we also studied the binding of ^3^H-dihydroalprenolol. There was no difference in the affinity of the receptors or the number of binding sites in the hippocampal membranes of the two genotypes (Table 2).

**Table 2:** Kinetic characteristics of^3^H-Citalopram,^ 3^H-Nisoxetine and^ 3^H-Dihydro-alprenolol binding in the hippocampus of P2rx7+/+ and P2rx7−/− mice.

	P2rx7+/+		P2rx7−/−	
	K_d_(nM)	B_max_(fmol/mg protein)	K_d_(nM)	B_max_(fmol/mg protein)
^3^H-citalopram(n = 4)	1.2±0.1	555±35	1.4±0.1	690±42*
^3^H-nisoxetine(n = 6)	0.7±0.05	126.8±4.9	0.7±0.1	131.4±7.5
^3^H-dihydroalprenolol(n = 3)	0.5±0.02	44.8±1.1	0.4±0.02	45.7±0.7

The number (B_max_) of ^3^H-Citalopram recognition sites is significantly increased in the hippocampus of P2rx7−/− mice compared with P2rx7+/+ mice (*P<0.05). For details of the procedures, see Methods. N = number of independent experiments.

## Discussion

Recent studies highlighted that P2X7 receptors play a regulatory role in a number of CNS-related functions, including learning and memory [55], sleep [56], fever [57] and behavior [23], [24], [25].

In our behavior experiments, we found that P2rx7−/− mice displayed an antidepressant-like phenotype in two tests, used to assess mood-related behavioral modifications pertinent to depression. In the TST model, an increased time of basal immobility was observed in the deficiency of P2rx7 consistent with the previous results obtained by our group [25] and of others using similar behavioral protocols [23], [24]. In our previous study, we also showed that similar changes could be reproduced by the pharmacological blockade of P2rx7 using the selective and *in vivo* active [58] P2rx7 antagonist Brilliant blue G. In the present study, we extended these data, showing that Brilliant blue G also counteracts bacterial endotoxin-induced depressive behavior, which suggests that P2rx7 regulates stress-induced depressive behavior.

Whereas the increased immobility in TST reflects the deficit in active coping strategy against negative environmental stimuli, another important aspect of depression is anhedonia, which is modeled in animal experiments by a decreased preference for sucrose in the SPT test. We have precipitated anhedonia in our experiments by a relatively mild endotoxin challenge, which elicited a selective decrease in sucrose, but not water consumption. Following the protocol, described by a previous study [41], the depression-like behavior can be temporally separated from the early symptoms of sickness behavior, such as the robust depression of motor activity and food intake [59]. Nevertheless, it should be noted that some behavioral, biochemical and electrophysiological changes persist days after LPS exposure and that even with this clear distinction LPS induced anhedonia response is only similar but not identical to depressive behavior characteristic to human major depression: whereas the former represents an adaptive response to infection, the latter occurs in the absence of immune challenge.

In our experiments, a significantly attenuated anhedonia response was observed in the deficiency of P2rx7, which could be pharmacologically reproduced using Brilliant blue G and another potent P2rx7 antagonist, AZ-10606120 [60]. Notably, AZ-10606120 was active upon acute application and displayed a higher potency than the potent antidepressant compound citalopram. Moreover, the effect of AZ-10606120, but not citalopram, was significantly attenuated in P2rx7−/− mice, confirming the involvement of P2rx7 in this effect. Interestingly, both AZ-10606120 and BBG was more effective in this test than the deletion of P2rx7. The compensatory upregulation of other P2X receptors, or the C-terminal truncated variant of P2rx7, which is not inactivated in the Pfizer-type knockouts [61] could be responsible for the discrepancy. In fact, we have previously showed the upregulation of P2rx4 in the striatum of the same P2rx7–deficient mouse line [62]. Another potential explanation is a P2rx7-independent component in the effect of antagonists. On the other hand, the relatively weak effect of acute citalopram treatment is consistent with previous literature data showing that anhedonia is largely sensitive to subacute or chronic, but not acute antidepressant treatment [63].

Overall, the behavioral data obtained in this study, together with previous results [23], [24], [25], provides further support for the hypothesis that P2X7 receptors regulate emotional behavior in animal models of depression.

To identify cellular actions that mediate the action of P2rx7 on mood-related behavior, we have chosen the hippocampus as the target area of our present studies. The rationale of the choice of hippocampus was two-fold: 1) a previous study [24] found that in parallel with a decreased response to repeated forced swimming test in P2rx7−/− animals a reduction in c-fos immunoreactivity was detected in the amygdala and hippocampus. 2) In our previous study P2rx7-dependent neurochemical alterations have already explored in the amygdala [25]. We have to note, however, that P2rx7-dependent changes in hippocampal signaling pathways found by the present study might also underlie other hippocampal functions such as memory formation. In fact, it has been described that there is a spatial memory deficit in the deficiency of P2rx7 [55], which was found to be correlated with impaired interleukin-1β and c-Fos expression in the hippocampus. On the other hand, non-hippocampal mechanisms might also participate directly or indirectly in the action of P2rx7 on mood-related behavior.

We examined glutamatergic transmission because one important function of P2rx7 activation in the brain is to promote the release of glutamate [19], [20] and previous observations from animal and human studies strongly indicate that excessive glutamatergic transmission might be involved in the pathophysiology of depressive disorders [64]. These findings include abnormalities in glutamate levels, e.g., [65], genetic polymorphism [66] and the differential expression of NMDA and AMPA receptor subunits in BPD and MDD patients [67], [68]; antidepressant properties of glutamate receptor antagonists and modulators [69]; and the regulation of glutamate receptor subunits by clinically used antidepressants [70], [71]. Moreover, in our previous study, consistent with the presumed dysfunction of glutamatergic transmission and consequent neuroplasticity changes in depression [69], [72], the downregulation of different AMPA and metabotropic glutamate receptor subunits and the upregulation of the NR2B subunit of NMDA receptors was detected in the amygdala of P2rx7−/− mice [25]. The real–time PCR analysis conducted in the present study revealed that the upregulation of the NR2B subunit of NMDA receptors also occurs in the hippocampus, which is another component of the limbic system. In addition, the results of the present study demonstrated that ATP elicits concentration-dependent tritiated and endogenous glutamate efflux in the hippocampus, and P2X7 receptors mediate the majority of this glutamate efflux, whereas a minor, residual portion of the glutamate release detected in the P2rx7−/− mice is mediated through P2X1 receptors. We also show that the basal [^3^H]Glu efflux is lower in the hippocampi of P2rx7 deficient animals, which is indicative for a lowered basal extracellular glutamate levels under the conditions of behavior experiments. These data are consistent with previous results showing that the activation of P2rx7 in the brain leads to increased glutamate release from the nerve terminals [19], [20], [22] and astrocytes [73], and both P2X7 and P2X1 receptors are expressed in the hippocampus [74]. Therefore the source of P2rx7 mediated glutamate release could be either neuronal or glial. Another possibility is that P2rx7 controls astrocytic glutamate uptake in the hippocampus; however this assumption needs further investigation.

A potential pathway, whereby increased glutamate release may lead to a change in mood is the alteration of the level of neurotrophic factors. Among them, BDNF is the most widely distributed neurotrophin in the CNS, and it plays several roles in synaptic plasticity and neuronal survival [75], [76]. The role of hippocampal BDNF and subsequent neurogenesis in depression and in the therapeutic action of antidepressants is an emerging hypothesis, which is supported by many experimental data [26], [77], [78], [79]. As a consequence, BDNF has become a key target in the pathology of several neurological and psychiatric diseases [80], and clinical studies have shown that BDNF protein expression is significantly decreased in both the serum and brain of depressed patients [29], [81], [82]. Therefore, we characterized the potential changes in BDNF expression in the hippocampus of wild type and P2rx7−/− mice. In our *ex vivo* study, we observed elevated basal BDNF levels in the hippocampus of P2rx7−/− mice compared with the corresponding saline-treated P2rx7+/+ controls. After systemic LPS challenge, we detected a decrease in BDNF protein expression in both genotypes. These results are consistent with previous data showing that hippocampal BDNF expression is significantly decreased at both the mRNA and protein levels in response to systemic LPS treatment [83], [84]. However, our results also imply that whereas an alteration in BDNF levels might be a mediator of P2rx7-dependent changes in behavior in the absence of LPS, e.g., the decreased basal immobility in the TST test (Fig. 1A), P2rx7-mediated glutamate efflux and subsequent changes in BDNF levels play only a minor role in the P2rx7-dependent regulation of LPS-induced depressive behavior (Fig. 1 B, C).

To explore the local regulatory role of P2X7 receptors in the modulation of basal BDNF production, we conducted an *in vitro* study. In support of the *ex vivo* results, the basal BDNF expression in the hippocampal slices of P2rx7−/− mice was significantly higher than in P2rx7+/+ mice, indicating a tonic inhibitory regulation of BDNF production through P2rx7. This was further confirmed using BBG, the selective P2rx7 antagonist; although BBG significantly increased the level of BDNF expression in both P2rx7+/+ and P2rx7−/− mice, its facilitatory effect was significantly attenuated in the deficiency of the P2X7 receptor. Interestingly, similar to our *in vivo* behavior studies, the pharmacological blockade of P2rx7 elicited a more pronounced effect than the genetic deletion, which could be explained by compensatory gene expression changes in case of genetic deletion.

When hippocampal P2rx7 was stimulated through the P2X receptor agonist BzATP, a significant decrease in BDNF protein expression was detected in P2rx7+/+ mice, which was absent in P2rx7−/− mice and reversed using BBG. These data indicate that BDNF levels in the hippocampus are under the local regulatory influence of P2rx7. To our knowledge, our study is the first to demonstrate a role for P2rx7 in the regulation of BDNF production in the central nervous system. In contrast, in the periphery, P2X7 receptor activation could induce the vesicular release of BDNF from the Schwann cells, which in turn might play a trophic role on neurons [85].

Because our previous data indicated that P2rx7 activation primarily releases glutamate [19], [20], we explored the role of glutamate receptors in the inhibitory action of BzATP on hippocampal BDNF protein expression. The inhibitory effect of BzATP was completely reversed by CNQX, the non-NMDA receptor antagonist TCN-201, the NR1/NR2A selective glutamate receptor antagonist and by the NR2B receptor selective antagonist RO-256981. These results indicate that the activation of both non-NMDA and NMDA receptors are necessary conditions for the P2rx7-mediated inhibitory regulation of BDNF level. Taken into account that glutamate receptor antagonists by themselves decreased BDNF production, consistently with the key role of synaptic glutamate receptor activation in the induction of BDNF [86], [87], a possible interpretation of our results is that glutamate released by P2rx7 activation primarily acted on extrasynaptic NMDA receptors, which are able to shut-off the induction of BDNF [86], but only if synaptic glutamate receptors are also co-activated. Because among NMDA receptor subunits, NR2B are primarily localized to extrasynaptic sites in the hippocampus [88], [89], [90] we propose that P2X7 receptor activation leads to increased glutamate release and to subsequent overactivation of extrasynaptic NR2B receptors. NR2B activation, in turn, downregulates BDNF expression and thereby causes long-lasting changes in neuronal plasticity, which might underlie pathological changes in behavior. The upregulation of hippocampal NR2B subunits after the genetic deletion of P2X7 receptors observed in these experiments also supports this proposed mechanism, consistent with the presumed dysfunction of glutamatergic transmission [91], [92] and consequent changes in neuroplasticity during depression [69], [72].

On the other hand, in the absence of exogenous activation of P2rx7 by BzATP, BDNF levels were decreased (CNQX, RO-258981) or unaffected (TCN-201) by ionotropic glutamate receptor antagonists, which implies that the net effect of endogenous P2rx7 activation and other signaling pathways converging on ionotropic glutamate receptors on BDNF level is stimulatory.

In contrast, effect of BzATP was not alleviated through the mGluR_1,5_ selective receptor antagonist, MCPG, whereas the administration of mGluR_1,5_ selective agonist DHPG, significantly increased BDNF expression in the presence and absence of BzATP. Therefore, mGluR_1,5_-mediated facilitatory modulation of hippocampal BDNF expression seems to be independent from the activation of P2rx7, consistently with the idea that BzATP-mediated glutamate release preferentially activates extrasynaptic NMDA receptors. Treatment with MCPG alone resulted in decreased BDNF expression, whereas DHPG treatment increased BDNF production, consistent with the well-known stimulatory role of mGluR_1,5_ receptors in BDNF production e.g. [93]. Interestingly, we also observed that BzATP paradoxically increased BDNF expression in the presence of the mGluR_1,5_ agonist, DHPG. This effect, which requires further investigation, is most likely independent from the activation of P2rx7.

In addition to the regulation of BDNF production, previous studies have associated neurogenesis with the beneficial actions of specific antidepressants, suggesting a connection between decreased hippocampal neurogenesis and depression [94], [95]. Others have hypothesized that neurogenesis might promote neuroplasticity [96], [97]. Our study revealed that basal level of neurogenesis detected using BrdU staining is higher in the deficiency of the P2X7 receptor in the dentate gyrus, suggesting that the endogenous activation of P2rx7 inhibits adult neurogenesis in the hippocampus through the regulation of BDNF levels or independently from it. Thus we cannot exclude the possibility that P2rx7 directly regulates hippocampal neuronal survival/neurogenesis without the involvement of glutamate or neurotrophic factors. Consistent with this latter assumption, the results of electrophysiological studies have shown that neuronal progenitor cells (NPCs) from the adult rat hippocampus [98], [99] and embryonic mouse striatum [100] express functional P2rx7. Activation of P2rx7 elicits necrotic cell death in the latter [100] and the inhibition of P2X7 receptors promotes axonal growth in cultured hippocampal neurons [101]. Therefore, further in situ analysis on the effect of the genetic deletion and pharmacological antagonism of P2rx7 on NPC survival and neurogenesis is of potential interest.

Taken together, the results obtained in this study provide several possible mechanisms to explain the antidepressant phenotype observed in the deficiency of P2rx7 [23], [24]. However, the signaling pathways mediating the effect of P2rx7 activation on stress-induced depressive behavior, i.e., those evoked through bacterial endotoxins, remains to be established. Thus, we determined whether neurochemical markers of monoaminergic transmission are changed in the hippocampus after genetic deletion of P2rx7 in saline and LPS-treated animals. Under basal conditions, we detected a dysregulation of monoaminergic transmission, and these results are consistent with our previous findings in the amygdala [25]. We observed elevated 5-HT levels and a decreased 5HIAA/5-HT ratio, reflecting the increased bioavailability of 5-HT, together with decreased basal NA levels. As an explanation for the elevated 5-HT levels, the number of [^3^H]Citalopram binding sites and the [^3^H]5-HT uptake was significantly higher in the hippocampus in the deficiency of P2rx7, whilst there was no difference in basal and stimulation-evoked [^3^H]5-HT release in the two genotypes. These findings are reminiscent of the effects of chronic antidepressant treatment, which increases the uptake of 5-HT in hippocampal slices [102]. However, we did not observe any change in NA transporter binding sites, and the ß-receptors were not downregulated, although it was previously published that treatment of rats with antidepressants reduced the number of ß-receptors, while serotonergic terminals remained intact [54]. The question arises whether the detected changes in serotonergic signaling in the hippocampus P2rx7 deficient mice are the cause or consequences of the alteration in glutamate and BDNF levels and neurogenesis, or parallel changes. The relationship between hippocampal serotonergic transmission, BDNF, neurogenesis and depressive behavior has been explored in numerous studies and there are several links between them. BDNF is critical for the normal development and function of central 5-HT neurons and the elaboration of behaviors that depend on the activity of these neurons [103], [104], [105]. As an example, elevation of extracellular 5-HT and antidepressant-like behavioral effects in the FST test by SSRI antidepressants are completely eliminated in mice deficient in BDNF (BDNF+/−) [104], [105] and a decrease in the [^3^H]5-HT uptake and [^3^H]citalopram binding densities are found in these mice [106], the opposite that is detected in P2rx7 deficient mice.

Interestingly, LPS treatment caused a significant elevation in 5-HT expression in the hippocampus of P2rx7+/+ mice, which was completely absent in P2rx7−/− mice; moreover, the 5-HT levels were depleted in these mice. Therefore, the direct or indirect modulation of serotonergic transmission is a potential mechanism that might underlie the action of P2rx7 to regulate stress-induced changes in mood-related behavior, although the possibility that alterations in 5-HT levels occur as a compensatory change in response to changes in behavior cannot be excluded.

Finally, it should be noted that although the present study focused on P2rx7, this is not the only pathway, whereby endogenous ATP or other purines could affect depressive behavior. ATP is a ubiquitous signaling molecule and the majority of its ionotropic (P2X1-7) and metabotropic (P2Y_1,2,4,6,11,12,13,14_) receptors are also expressed in the hippocampus and other brain regions involved in the processing of depressive behavior. The identification of the role of these receptors, however, awaits further investigation.

In conclusion, our data show that the genetic deletion of P2rx7 leads to an antidepressant phenotype associated with changes in hippocampal monoaminergic transmission, neurotrophin protein expression and adult neurogenesis in the dentate gyrus. Moreover, these data support the view that P2rx7 antagonists might have therapeutic potential in mood-related disorders.
